# Siroheme Is Essential for Assimilation of Nitrate and Sulfate as Well as Detoxification of Nitric Oxide but Dispensable for Murine Virulence of *Aspergillus fumigatus*

**DOI:** 10.3389/fmicb.2018.02615

**Published:** 2018-11-12

**Authors:** Anna-Maria Dietl, Ulrike Binder, Yana Shadkchan, Nir Osherov, Hubertus Haas

**Affiliations:** ^1^Division of Molecular Biology, Biocenter, Medical University of Innsbruck, Innsbruck, Austria; ^2^Division of Hygiene and Medical Microbiology, Medical University of Innsbruck, Innsbruck, Austria; ^3^Department of Clinical Microbiology and Immunology, Sackler School of Medicine, Tel-Aviv University, Tel-Aviv, Israel

**Keywords:** *Aspergillus fumigatus*, virulence, siroheme, sulfate and nitrate assimilation, nitric oxide detoxification

## Abstract

The saprophytic mold *Aspergillus fumigatus* is the most common airborne fungal pathogen causing severe invasive fungal infections in immunocompromised patients. Siroheme is a heme-like prosthetic group used by plants and microorganisms for sulfate and nitrate assimilation but is absent in higher eukaryotes. Here, we investigated the role of siroheme in *A. fumigatus* by deletion of the gene encoding the bifunctional dehydrogenase/ferrochelatase enzyme Met8. Met8-deficiency resulted in the inability to utilize sulfate and nitrate as sulfur and nitrogen sources, respectively. These results match previous data demonstrating that siroheme is an essential cofactor for nitrite and sulfite reductases. Moreover, Met8-deficiency caused significantly decreased resistance against nitric oxide (NO) underlining the importance of nitrite reductase in NO detoxification. Met8-deficiency did not affect virulence in murine models for invasive aspergillosis indicating that neither NO-detoxification nor assimilation of sulfate and nitrate play major roles in virulence in this host. Interestingly, Met8-deficiency resulted in mild virulence attenuation in the *Galleria mellonella* infection model revealing differences in interaction of *A. fumigatus* with *G. mellonella* and mouse.

## Introduction

*Aspergillus fumigatus* is a major fungal pathogen causing a wide range of invasive and non-invasive infections. While a healthy human immune system is able to efficiently eliminate daily inhaled *A. fumigatus* spores, in immunocompromised patients, conidia are able to germinate in the alveoli and subsequently cause the life-threatening disease invasive aspergillosis (Brakhage and Langfelder, 2002; Tekaia and Latge, 2005; Dagenais and Keller, 2009). Treatment possibilities for invasive fungal infections remain limited, due to the fact that fungi are eukaryotes and consequently share the majority of metabolic pathways with mammals. In the search for new antifungal targets, taking into account protein domains and domain architecture, the cofactor siroheme was identified as a potential target for antifungal drugs as it is absent in mammals (Barrera et al., 2014). Here, we characterized the role of the heme-like tetrapyrrole siroheme in *A. fumigatus*. In plants, bacteria and *Saccharomyces cerevisiae*, siroheme has been shown to be essential for sulfate assimilation as cofactor for sulfite reductase (Crane and Getzoff, 1996; Hansen et al., 1997; Raux et al., 1999). In fact, siroheme is essential for life on earth, as reduction of sulfite to sulfide is the prerequisite for incorporation of sulfur into organic molecules (Tripathy et al., 2010). Furthermore, in plants and bacteria, siroheme has been shown to be essential for nitrate assimilation as it serves as cofactor for nitrite reductase. Most fungal species employ nitrate assimilation but the model system *S. cerevisiae* lacks this nitrogen assimilation system. In agreement with a role in fungal nitrate assimilation, the nitrite reductase from *Neurospora crassa* was shown biochemically to contain siroheme as prosthetic group (Schubert et al., 2002). Mammals are incapable of reduceing sulfate or nitrate and satisfy their need for sulfur and nitrogen by uptake of organic sources such as amino acids from their diet (Tripathy et al., 2010). The major mammalian sulfur source is methionine, while cysteine is non-essential and a metabolite of methionine metabolism (Townsend et al., 2004).

**FIGURE 1:**
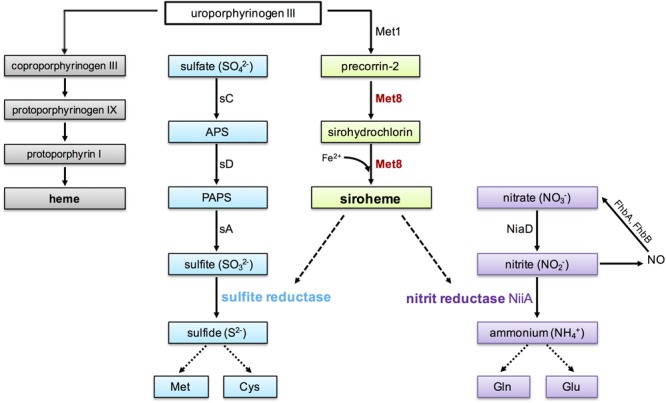
Biosynthesis of siroheme and heme from uroporphyrinogen III and assimilation of sulfur and nitrogen in *Aspergillus fumigatus*. Heme biosynthesis is shown in gray, siroheme biosynthesis in green, assimilation of sulfate in blue and nitrate, as well as NO detoxification is shown in purple. The bifunctional protein Met8 (precorrin-2 dehydrogenase/siroheme ferrochelatase, AFUA_7G05680), investigated in the current study is highlighted in red. Dashed lines demonstrate the requirement of siroheme as cofactor for the β-subunit of sulfite reductase (AFUA_2G15590/homolog of *Saccharomyces cerevisiae* Met5) and nitrite reductase (AFUA_1G12840/NiiA), respectively. Notably, sulfite reductase has a second subunit (AFUA_6G08920/sF). Nitric oxide (NO) radicals are detoxified via nitrite by the cytosolic flavohemoglobin FhpA (AFUA_4G03410) and the mitochondrial flavohemoglobin FhpB (AFUA_8G06080). APS: adenosine 5′-phosphosulfate; PAPS: 3′-phosphoadenosine-5′-phosphosulfate; Met1: uroporphyrin-III C-methyltransferase (AFUA_3G06600); sC: ATP-sulfurylase (AFUA_3G06530); sD: APS kinase (AFUA_1G10820); sA: PAPS reductase (AFUA_3G06540).

Siroheme derives from the heme biosynthetic pathway and is synthesized from the common intermediate uroporphyrinogen III in three enzymatic reactions (Figure 1, gray and green boxes): methylation to precorrin-2, dehydrogenation to sirohydrochlorin, and incorporation of ferrous iron by a ferrochelatase to generate siroheme (Raux et al., 1999). In *Escherichia coli* and some other bacteria, all three steps are performed by a single multifunctional enzyme named CysG (Warren et al., 1990, 1994; Spencer et al., 1993), while in *S. cerevisiae* these steps are performed by two enzymes, termed Met1p and Met8p. Met1p catalyzes the methylation reaction whereas the bifunctional enzyme Met8p performs both the dehydrogenation and ferrochelation reactions (Raux et al., 1999).

Fungi can utilize organic sulfur sources such as sulfur-containing amino acids or inorganic sulfur sources such as sulfate. Sulfate assimilation in *A. fumigatus* (summarized in Figure 1, blue boxes) starts with cellular uptake of sulfate (SO_4_^2-^) by the sulfate transporter and reduction into sulfite (SO_3_^2-^) requiring three enzymes: the ATP sulfurylase (sC), adenosine 5′-phosphosulfate (APS) kinase (sD), and 3′-phosphoadenosine-5′-phosphosulfate (PAPS) reductase (sA). Subsequently, sulfite is reduced to sulfide by sulfite reductase (Amich et al., 2016). Sulfide is an essential component for primary and secondary metabolism such as production of the proteinogenic amino acids methionine and cysteine, the coenzyme A or the antioxidant glutathione (Leustek et al., 1997; Lawlor, 2002; Tripathy et al., 2010).

With some exceptions such as *S. cerevisiae*, most fungal species including *A. fumigatus* are not only able to utilize ammonium, amino acids, and purines but also nitrate as nitrogen source (Marzluf, 1993). The first step in nitrate assimilation in *A. fumigatus* is the cellular uptake of nitrate mediated by a nitrate transporter (CrnA) followed by the step-wise reduction of nitrate (NO_3_^-^) by nitrate reductase (NiaD) and nitrite (NO_2_^-^) reductase (NiiA) to ammonium (NH_4_^+^) (Pateman et al., 1967; Johnstone et al., 1990). A scheme is shown in Figure 1 (purple boxes). Ammonium is then incorporated into the nitrogen pool via conversion of glutamate into glutamine.

On the one hand, nitrate assimilation generates nitric oxide (NO) as a byproduct (Schinko et al., 2010). On the other hand, the nitrite reductase involved in nitrate assimilation is crucial for detoxification of exogenous NO sources. NO and reactive nitrogen radicals are for example used by the immune system to attack invading pathogens (Fang, 1997; Brown et al., 2009). Cellular NO detoxification is mediated by conversion of NO to nitrate via flavohemoglobins (FhpA and FhpB) and decomposition via nitrate reductase and nitrite reductase to ammonium (Lapp et al., 2014). A scheme is shown in Figure 1. Consequently, inactivation of nitrite reductase is expected to increase cellular NO stress due to increased accumulation of nitrite, which again spontaneously decomposes to NO. Additional cellular NO detoxification mechanisms include reduction of *S*-nitrosoglutathione to ammonium and glutathione by a *S*-nitrosoglutathione reductase (GnoA) and NO-removal by NO-inducible nitrosothionein (NtpA) in concert with thioredoxin and thioredoxin reductase (Zhou et al., 2013). Nevertheless, loss of flavohemoglobin-mediated NO detoxification cannot be compensated by the alternative detoxification strategies (Lapp et al., 2014).

Taken together, the impact of lacking siroheme biosynthesis on fungal physiology has so far only been studied with respect to sulfate assimilation in *S. cerevisiae*. In this study, we investigated the role of siroheme in sulfate assimilation, nitrate assimilation, NO detoxification and virulence in *A. fumigatus*.

## Materials and Methods

### Strains, Media and Growth Conditions

*Aspergillus fumigatus* strains were generally cultured in *Aspergillus* minimal medium according to Pontecorvo et al. (1953), containing 1% glucose as carbon source and 20 mM L-glutamine or 70 mM NaNO_3_ as nitrogen source, respectively, or in complex medium containing 2 g/L peptone and 1 g/L yeast extract, trace elements and salts (Pontecorvo et al., 1953) at 37 or 30°C. When indicated, media were supplemented with L-methionine, taurine, L-cysteine, sodium sulfide nonahydrate, hemin chloride or sodium nitrite. *Aspergillus* minimal medium contains 2.2 mM sulfate, mainly derived from MgSO_4_ and trace element solutions. Blood agar contained 1.8% agar, 0.5% sodium chloride, and 10% blood. Agar plates with homogenized *Galleria mellonella* extracts or hemolymph of *G. mellonella* contained 1.8% agar, 0.5% sodium chloride, 160 mg/L gentamycin, and 10% *G. mellonella* extract or 10% hemolymph, respectively. For the extract, one frozen *G. mellonella* (∼0.5 g) was homogenized by mixing with glass beads and dissolved in 1.0 ml insect physiological saline (IPS; 150 mM NaCl, 5 mM KCl, 10 mM EDTA, and 30 mM sodium citrate in 0.1 M Tris–HCl, pH 6.9). Conidia for pulmonary mouse infection were cultivated on solid YAG medium (0.5% yeast extract, 1% glucose, 10 mM MgCl_2_, trace elements, and vitamin solution). Conidia for the insect infection model were cultivated on solid minimal medium with 20 mM glutamine and 1 mM methionine as nitrogen and sulfur sources respectively. Liquid cultures were inoculated with 10^6^ conidia/ml medium and incubated at 37°C for 24 h. For quantification of biomass production, mycelia from liquid cultures were freeze-dried and weighed. For growth assays, 10^4^ spores were point-inoculated on plates for 48 h at 37°C. As recipient strain for genetic manipulation of *A. fumigatus*, the *akuA-*deficient derivative of ATCC46645, AfS77, termed wild type (wt) here, was used (Krappmann et al., 2006).

### Deletion of Δ*met8* (AFUA_7G05680) and Reconstitution of the Δ*met8* Strain

**FIGURE 2:**
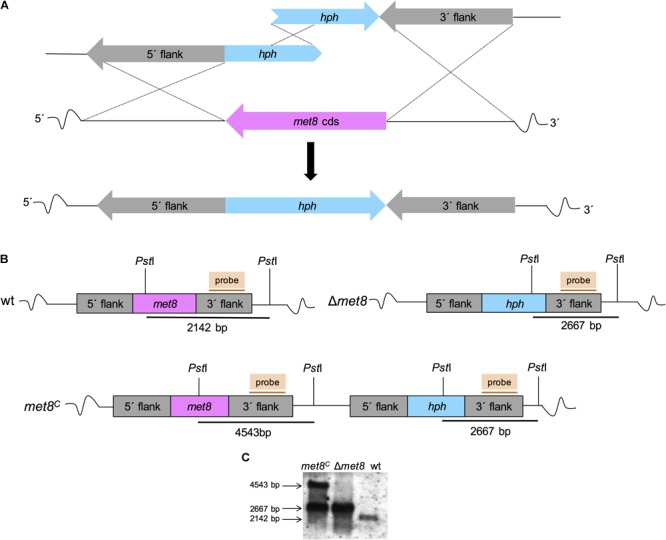
Deletion and reconstitution of the *met8* gene in *A. fumigatus*. **(A)** Schematic view of the split-marker technique-mediated deletion of *met8* in *A. fumigatus* wt AfS77. **(B)** Genomic organization of the *met8* locus in wt, Δ*met8* and *met8^C^.* Genomic DNA digestion with *PstI* resulted in a 2.1-kb for the wt, a 2.6-kb for Δ*met8* and a 2.6-kb and 4.5-kb fragment for *met8^C^*, respectively. **(C)** Southern blot analysis of genomic DNA of wt, Δ*met8* and *met8^C^* using hybridization probes indicated in **(B)** confirming the genetic manipulation.

The *met8* coding sequence was deleted in AfS77 using the bipartite marker technique (Nielsen et al., 2006). Therefore, the *A. fumigatus* strain AfS77 was transformed with two DNA constructs containing 5′- and 3′- incomplete but overlapping fragments of the hygromycin resistance cassette (*hph*) fused to 972 bp (using primer oAfmet8-1f and oAfmet8-2r) and 926 bp (using primer oAfmet8-4f and oAfmet8-5r) of *met8* flanking sequences, respectively (Figure 2). The fragments were digested with *Avr*II and *Xba*I, respectively, and ligated to the *hph* selection marker released from plasmid pan7.1 (Punt et al., 1987) by digestion with *Avr*II and *Xba*I. The ligation product served as template for the two overlapping fragments, amplified with primer oAfmet8-3nested and ohph14 (2189 bp) for the 5′- flanking region and oAfmet8-6nested and ohph15 (2355 bp) for the 3′- flanking region. The flanking regions shared a 447 bp overlap with the *hph* cassette to serve as recombination site during transformation. Δ*met8* transformants were selected with 0.1 mg⋅ml^-1^ hygromycin B (Calbiochem) on minimal medium plates.

For reconstitution of Δ*met8*, a functional *met8* copy was subcloned into the pGEM-T Easy (Promega) plasmid. Taking advantage of siroheme auxotrophy of the Δ*met8* mutant, protoplasts were transformed with *pmet8* (linearized with *Nhe*I, localized in the 5′flank of *met8* and thereby promoting homologous recombination in the 5′flank), yielding the complemented strain *met8^C^* (Figure 2). Correct genetic manipulation of transformants was confirmed by Southern blot analysis. Genomic DNA from mycelia was isolated according to Sambrook et al. (1989). Primers used for the genetic manipulation of the mutant strains (Table 1) are listed in Table 2.

**Table 1:** Fungal strains used in this study.

Strain	Description	Reference
AfS77 (wt)	ATCC46645*, akuA*(AFUA_5G05680)*::loxP*	Krappmann et al., 2006
Δ*met8*	*met8*(AFUA_7G05680)*::hph;* AfS77	This study
*met8^C^*	*met8::hph;* AfS77*; met8*	This study

**Table 2:** Primers used for generation of Δ*met8 and met8^C^.*

Primer	Sequence 5′-3′
oAfmet8-1f	CAC ATC ACG CAC ACG CAC
oAfmet8-2r	TGC GAG **CCT AGG** CCA TCC TGT CCT TGC TGA G
oAfmet8-4f	TTT GTT **TCT AGA** ACA TTC AAC ACT CCT CCA G
oAfmet8-5r	GCC GAA CCT CAA CAG CAG
oAfmet8-3nested	GAG CAG CGG GTG GTG TC
oAfmet8-6nested	GCG TGC TTC AAC TAC TTA TG
ohph15	GAG AGC CTG ACC TAT TGC
ohph14	TCT CGT CTT CCT CAT TCT C

*Add-on restriction enzyme sites for *Avr*II and *Xba*I, respectively, are marked in bold.*

### *Galleria mellonella* Infection Studies

*Galleria mellonella* virulence studies were carried out according to Maurer et al. (2015). Sixth instar larvae (K. Pechmann, Biologische Wurmzucht, Langenzersdorf, Austria) were kept at 18°C in the dark before use. 1 × 10^7^ *A. fumigatus* conidia were suspended in 20 μl insect physiological saline (IPS) and injected into the hemocoel via one of the hind pro-legs. Infected *G. mellonella* larvae were incubated at 30°C in the dark and survival was monitored daily up to 6 days. To avoid temperature-triggered effects on the larval immune response, incubation was favored at 30°C (Mowlds and Kavanagh, 2008). Survival data were evaluated by Kaplan Meier curves and significance determined with log-rank (Mantel-Cox) test, utilizing GraphPad Prism 7.00 software. Differences were considered significant at *P*-values ≤ 0.05.

### Pulmonary Mouse Infection

Two immunocompromised murine models for pulmonary aspergillosis were used: for the (i) non-neutropenic model (CA model), 6-week-old female ICR mice were immunocompromised by subcutaneous injection with cortisone acetate (300 mg/kg) 3 days prior to infection, on the day of infection, and 3, 7, and 11 days post-infection. Inocula were prepared by harvesting conidia from 3-day-old solid YAG cultures. 5 × 10^5^ dormant spores were suspended in 20 μl of PBS with 0.2% Tween 20 and injected intranasally (10 μl in each nostril). For the (ii) neutropenic model (CY model), 6-week-old female ICR mice were immunocompromised with cyclophosphamide (150 mg/kg in PBS) injected intraperitoneally 3 days prior and 2 days post-conidial infection. In addition, 3 days prior to conidial infection, cortisone acetate (150 mg/kg in PBS) was injected subcutaneously. Disease progression and survival was monitored for up to 21 days. The statistical differences for mouse survival were calculated using the log-rank (Mantel-Cox) test. Differences were considered significant at *P*-values ≤ 0.05. This study was carried out in accordance with the recommendations of the ministry of Health (MOH) Animal Welfare Committee, Israel. The protocol was approved by the MOH Animal Welfare Committee, Israel.

## Results

### Generation of an *A. fumigatus* Mutant Strain Lacking Siroheme

To analyze the role of siroheme in *A. fumigatus*, we deleted the gene *met8* (AFUA_7G05680) encoding the homolog of *S. cerevisiae* Met8p (precorrin-2 dehydrogenase/ferrochelatase), a key enzyme of the siroheme biosynthetic pathway, by replacing the coding region with the hygromycin resistance cassette (*hph*) as described in the section “Material and Methods” and Figure 2. The *A. fumigatus akuA::loxP* strain derived from ATCC46645 (AfS77, termed wt here), largely lacking non-homologous recombination (Krappmann et al., 2006; Hartmann et al., 2010), was used as recipient strain. The *met8* deletion mutant (termed Δ*met8*) was complemented (termed *met8^C^*) with a *met8* copy inserted upstream of the deletion locus to ascertain *met8*-specific effects. Preliminary phenotypical analysis demonstrated an inability of Δ*met8* to grow on minimal medium with glutamine as sole nitrogen source and sulfate as sole sulfur source. This enabled re-integration of a functional *met8*-gene copy at its original locus with selection for growth with sulfate as sulfur source without an additional selection marker as described in the section “Material and Methods” and Figures 2A,B. Correct genetic manipulation was confirmed by Southern blot analysis (Figure 2C).

### Lack of Met8 Impairs Assimilation of Sulfate and Nitrate

**FIGURE 3:**
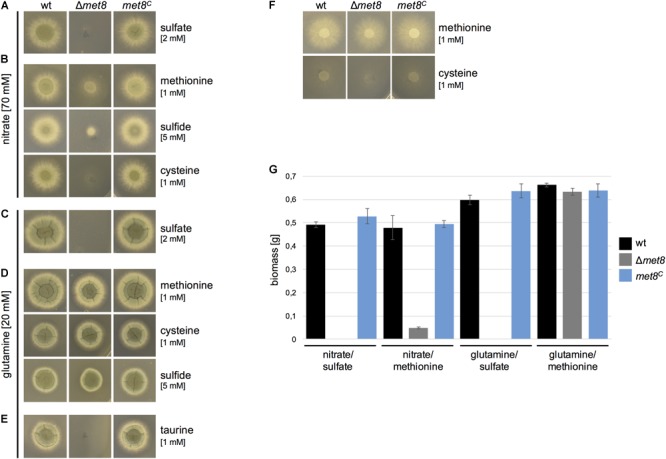
Deletion of *met8* in *A. fumigatus* impairs assimilation of nitrate and sulfate. **(A–E)** 10^4^ spores of the respective fungal strains were point-inoculated on minimal medium supplemented with either nitrate or glutamine as *N*-source and sulfate, taurine, sulfide, cysteine or methionine as *S*-source, respectively. In **(F)**, methionine or cysteine served as both sulfur and nitrogen source. Photographs were taken after 48 h of incubation at 37°C. **(G)** Biomass production (dry weight) of wt, Δ*met8* and *met8^C^* was quantified after growth for 22 h in liquid minimal medium. Cultures were supplemented as indicated with either 70 mM nitrate, 20 mM glutamine and/or 5 mM methionine. Data represent the mean of three biological replicates ± standard deviation. Minimal medium contains 2.2 mM sulfate.

To analyze if siroheme is indeed an essential cofactor for assimilation of sulfate and nitrate, growth of Δ*met8* was tested on solid minimal media with various nitrogen and sulfur sources (Figure 3). Compared to the wt and *met8^C^*, Δ*met8* was unable to grow in the presence of nitrate (NO_3_^-^) and sulfate (SO_4_^2-^) as sole sulfur and nitrogen sources, respectively, on agar plates (Figure 3A) and in liquid cultures (Figure 3G).

In a next step, the growth on nitrate as nitrogen source combined with organic or reduced sulfur sources was tested (Figure 3B). Nitrate as nitrogen source in combination with methionine as sulfur source allowed only limited growth of Δ*met8* on plates (Figure 3B) and in liquid cultures (Figure 3G). This limited growth can be explained by the fact that methionine can serve as both sulfur and poor nitrogen source because methionine without an additional nitrogen source allowed poor growth without sporulation on plates, whereby wt, *met8^C^*, and Δ*met8* displayed the same phenotype (Figure 3F). Moreover, chemical decomposition of nitrate cannot be excluded (Mu and Perlmutter, 1982). Nitrate as nitrogen source in combination with sulfide (reduced sulfur) as sulfur source also failed to rescue the growth defect of Δ*met8* (Figure 3B). In combination with nitrate, cysteine promoted growth to a lesser extent compared to methionine (Figure 3B), most likely because it performed worse as sole nitrogen and sulfur source compared to methionine (Figure 3F).

Glutamine as nitrogen source in combination with sulfate as sulfur source also failed to rescue growth on plates (Figure 3C) or in liquid cultures (Figure 3G).

In contrast, the combination of glutamine and methionine as sulfur source rescued wt-like biomass formation in liquid growth (Figure 3G) and almost wt-like colony formation on plates (Figure 3D). Similarly, the combination of glutamine with either cysteine or sulfide as sulfur source yielded wt-like growth on agar plates (Figure 3D).

Taken together, these results indicate that siroheme is indeed crucial for assimilation of sulfate and nitrate in *A. fumigatus* and, based on the literature, this is due to requirement of siroheme as cofactor for sulfite reductase and nitrite reductase, respectively. In other words, lack of siroheme does not affect growth on nitrogen sources not requiring nitrate assimilation (such as glutamine) in combination with organic sulfur sources (e.g., methionine, cysteine) or reduced sulfur (sulfide) (Figures 3D,G).

The organic compound taurine is a byproduct of the metabolism of the sulfurous amino acids cysteine and methionine and has been shown to be widely distributed in the bronchoalveolar lavage fluid of both healthy and asthmatic patients (Hofford et al., 1997). Many bacterial and fungal species, including *A. fumigatus*, are able to utilize taurine as a sulfur source (Amich et al., 2013). The Δ*met8* mutant strain was not able to utilize taurine as sulfur source (Figure 3E). Most likely, taurine/α-ketoglutarate dioxygenase releases sulfite from taurine, which must be further reduced via sulfite reductase to sulfide to become incorporated into organic sulfur compounds (Hogan et al., 1999). In agreement, lack of sulfite reductase was shown to block utilization of taurine in *A. fumigatus* (Amich et al., 2016).

**FIGURE 4:**
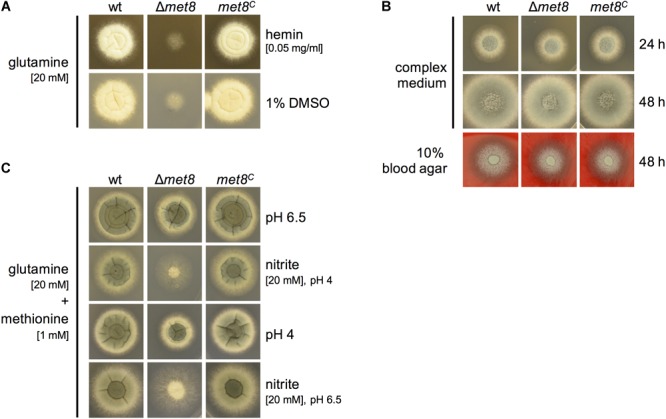
Lack of siroheme cannot be cured by hemin supplementation **(A)**, lack of Met8 does not affect growth in complex medium or on blood agar **(B)** and siroheme plays an important role in NO detoxification **(C)**. 10^4^ spores of the respective fungal strains were point-inoculated on the indicated growth medium and photographs were taken after 48 h of incubation at 37°C. **(A)** Minimal medium was supplemented with glutamine as *N*-source and 0.05 mg/ml hemin. Addition of 1% DMSO serves as growth control. **(C)** Minimal medium was supplemented with glutamine as *N*-source and methionine as *S*-source and adjusted to pH 6.5. As a control, this medium was alternatively adjusted to pH 4. To induce NO stress, 20 mM nitrite was added and the medium was adjusted to pH 4 or pH 6.5.

To analyze whether hemin is able to compensate the lack of siroheme biosynthesis, we analyzed growth of Δ*met8* on minimal medium in the presence of 0.05 mg/ml hemin, dissolved in 1% DMSO with glutamine as nitrogen source. The wt and *met8^C^* displayed a sporulation defect due to the presence of 1% DMSO in which hemin is dissolved. Remarkably, the sulfoxide containing DMSO enabled limited growth of Δ*met8* (Figure 4A) indicating that it serves as a poor sulfur source. Hemin did not further improve the growth of Δ*met8* revealing that hemin cannot compensate the lack of siroheme. Complex medium, which contains organic sulfur and nitrogen compounds from yeast extract, casamino acids, and peptone, allowed full growth Δ*met8* (Figure 4B). Similarly, Δ*met8* showed wt-like growth on 10% blood, indicating that blood contains sufficient reduced sulfur and nitrogen sources (Figure 4B).

### Lack of Met8 Decreases Resistance Against Nitric Oxide (NO)

One possibility for NO detoxification involves conversion to nitrate by flavohemoglobins followed by degradation of nitrate to nitrite and ammonium catalyzed by nitrate reductase and nitrite reductase (Lapp et al., 2014). To analyze the role of siroheme/nitrite reductase in NO detoxification of *A. fumigatus*, Δ*met8*, wt, and *met8^C^* were subject to NO stress by growth on minimal medium with glutamine as nitrogen source and methionine as sulfur source with addition of 20 mM sodium nitrite (NaNO_2_) at pH 4 because nitrite is known to decompose to NO particularly under acidic conditions (Schinko et al., 2010). In this setup, lack of Met8 caused significantly decreased growth (Figure 4C), indicating the importance of siroheme and consequently nitrite reductase in NO detoxification. Two lines of evidence emphasize that the growth inhibiting effect is indeed mediated by NO: (i) pH 4 in the absence of nitrite had only a minor effect on the growth of Δ*met8* and (ii) the growth reduction caused by nitrite was significantly lower at pH 6.5 compared to pH 4.

### Lack of Met8 Results in Virulence Attenuation of *A. fumigatus* in the Insect Host Model *Galleria mellonella*

**FIGURE 5:**
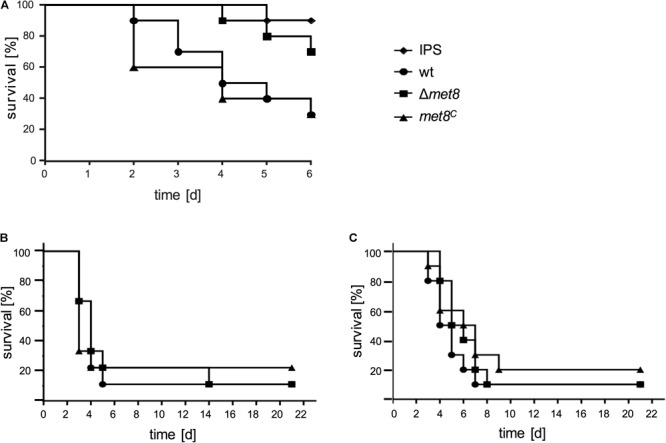
Lack of Met8 affects virulence of *A. fumigatus* in the insect model *Galleria mellonella* **(A)** but not in murine models for invasive aspergillosis **(B,C)**. **(A)** *G. mellonella* larvae were infected with conidia of the wt, Δ*met8* and *met8^C^* and survival was monitored over a period of 6 days. Control cohorts were injected with the conidial solution buffer (IPS). Met8-deficiency (Δ*met8*) attenuates virulence of *A. fumigatus* in *G. mellonella* compared to wt and *met8^C^* strain (*P* = 0.0365 vs. wt; *P* = 0.0258 vs. *met8^C^*). Survival curve for mice following intranasal infection of **(B)** cortisone-acetate immunocompromised mice (wt, *n* = 9; Δ*met8, n* = 9; *met8^C^, n* = 9 animals/group) and **(C)** cyclophosphamide-immunocompromised neutropenic mice (wt, *n* = 10; Δ*met8, n* = 10; *met8^C^, n* = 10 animals/group). Infection with Δ*met8* shows wt-like virulence in both murine models.

To analyze the role of siroheme in terms of pathogenicity wt, Δ*met8*, and *met8^C^* were compared in the *G. mellonella* infection model. Deletion of *met8* resulted in a significantly higher survival rate of *G. mellonella* larvae compared to larvae infected with the wt and *met8^C^* strain over a period of 6 days. After 4 days, only 50% of *G. mellonella* larvae infected with the wt and 40% with the *met8^C^* strain, respectively, survived whereas 90% of larvae infected with Δ*met8* were still alive (Figure 5A). The attenuated virulence of the *met8*-deletion mutant indicates that nitrate and/or sulfate assimilation, or the defect in NO detoxification due to loss of siroheme biosynthesis plays an important role for virulence of *A. fumigatus* in the insect model.

### Lack of Met8 Does Not Affect Virulence of *A. fumigatus* in a Murine Model of Invasive Aspergillosis

To assess the role of siroheme in murine bronchopulmonary aspergillosis, nine 6-week-old ICR mice per group were immunocompromised with cortisone acetate (CA, non-neutropenic host model), and intranasally infected with 5 × 10^5^ spores of the wt, Δ*met8* or *met8^C^*. As shown in Figure 5B, survival curves demonstrate that infection with all three strains caused comparable high mortality rates (e.g., at 7 days post-infection: wt, 90%; Δ*met8*, 80%; *met8*^C^, 80%).

In addition, virulence of wt, Δ*met8* or *met8^C^* was compared in a neutropenic infection model in which cyclophosphamide (CY) was used for immunosuppression. 5 × 10^5^ spores of the respective strains were intranasally infected, and survival was monitored for up to 21 days (Figure 5C). Mice infected with Δ*met8* showed similar mortality rates to mice infected with the wt and *met8^C^* (e.g., at 7 days post-infection: wt, 90%; Δ*met8*, 80%; *met8^C^*, 70%). This result is supported by the wt-like growth of Δ*met8* on blood agar (Figure 4B). Taken together, in these two different murine models for invasive aspergillosis, lack of siroheme biosynthesis did not affect the virulence of *A. fumigatus* demonstrating that neither sulfate assimilation, nor nitrate assimilation or NO detoxification play major roles in virulence of *A. fumigatus* in the mouse model.

### Lack of Met8 Does Not Affect Growth on *G. mellonella* Extracts and Impacts Growth Similarly at 30 and 37°C

**FIGURE 6:**
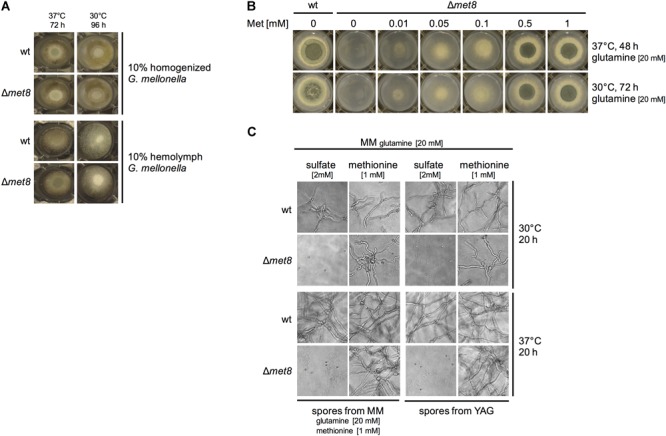
Lack of Met8 does not affect growth on *G. mellonella* extracts and hemolymph **(A)**, does not impact growth at 30°C compared to 37°C **(B)**, and blocks germination in minimal medium lacking an organic sulfur source **(C)**. **(A,B)** 10^4^ spores of the respective fungal strain were point-inoculated on the indicated growth medium and photographs were taken after the indicated incubation period and temperature. **(C)** To analyze germination and growth, 10^5^ conidia/ml were inoculated in liquid minimal medium containing glutamine as nitrogen source and either sulfate or methionine as sulfur source; photographs were taken after the indicated incubation period and temperature.

The data presented above revealed that lack of Met8 attenuates virulence in the insect model but not in the murine models. Possible reasons might be differences in nitrogen and sulfur sources in the two host niches or the difference in the temperature as the insect model was conducted at 30°C while the mouse host temperature is 37°C. Therefore, we compared the growth of the Δ*met8* mutant strain compared to wt on solid agar containing either 10% homogenized *G. mellonella* extract or 10% *G. mellonella* hemolymph at 30 and 37°C, respectively. The Δ*met8* mutant and the wt strain displayed comparable growth on both media at both temperatures (Figure 6A).

To further analyze if the growth of the Δ*met8* mutant strain is temperature dependent, we analyzed its growth at 30 and 37°C with glutamine as nitrogen source in the presence of increasing methionine concentrations. Figure 6B shows the comparison of incubation at 30°C for 72 h with incubation at 37°C for 48 h. These different incubation times were chosen to compensate for the slower growth at 30°C and as the wt (as well as the Δ*met8* mutant with high methionine supplementation) displayed a similar radial growth at these two conditions. The similar growth pattern of the Δ*met8* mutant with limiting methionine supplementation at 30 and 37°C indicates that the growth of the Δ*met8* mutant is not temperature dependent.

Conidia used for the murine infection models were generated on YAG medium, while conidia used for the insect model were generated on minimal medium with glutamine and methionine as nitrogen and sulfur sources, respectively. Figure 6C shows that Δ*met8* conidia are not able to germinate at 30 or 37°C on minimal medium containing glutamine as nitrogen source and sulfate as sulfur source independent from the conidia production medium, YAG or minimal medium. In contrast, Δ*met8* conidia from both media germinate and form hyphae on minimal medium with methionine as sulfur source. Moreover, wt conidia from both media germinate and form hyphae on minimal medium with sulfate as well as with methionine as sulfur source. These data indicate that neither YAG nor minimal medium with methionine as sulfur source is able to generate a conidial organic sulfur depot being sufficient for germination or lead to contamination with organic sulfur enabling germination. Taken together, these data argue against an impact of the growth medium on germination of Δ*met8* conidia. Moreover, these data demonstrate that an exogenous organic sulfur source is essential for germination of Δ*met8* conidia.

## Discussion

This study represents the first functional analysis of the role of siroheme in a fungal species employing assimilation of both sulfate and nitrate and the first analysis of its role in virulence. Among fungi, so far only the role of siroheme in sulfate assimilation by *S. cerevisiae* had been studied (Hansen et al., 1997; Raux et al., 1999). The only siroheme-dependent enzymes known are sulfite reductase and nitrite reductase (Murphy et al., 1974). Our data demonstrate that these functions are conserved in *A. fumigatus* as lack of the siroheme biosynthetic enzyme Met8, and consequently siroheme biosynthesis, impaired assimilation of both sulfate and nitrate assimilation (Figures 3A–C). In agreement, both nitrite reductase (AFUA_1G12840, NiiA) and the β-subunit of sulfite reductase (AFUA_2G15590, homolog of *S. cerevisiae* Met5) of *A. fumigatus* contain the siroheme consensus binding motif [STVN]-G-C-X_3_-C-X_6_-[DE]-[LIVMF]-[GAT]-[LIVMF] (PROSITE NIR_SIR, PS00365^1^; X represents any amino acid residue), i.e., ^758^**SGC**VRE**C**AEAQNK**DFGL** in nitrite reductase and ^1418^**TGC**PNG**C**ARPWLA**EVAF** in sulfite reductase. The wt-like growth of the Met8-lacking mutant with glutamine as nitrogen source and methionine as sulfur source, which makes assimilation of both sulfate and nitrate dispensable, did not indicate additional siroheme-dependent pathways in *A. fumigatus*. This is underlined by wt-like virulence in the murine infection model, which represents a complex growth niche. In agreement, a search of the *A. fumigatus* proteome for proteins containing the siroheme binding motif (PROSITE NIR_SIR, PS00365^1^) using FIMO^2^, identified only the nitrate and sulfite reductases (Grant et al., 2011).

Moreover, we demonstrate that Met8 deficiency impairs NO detoxification, most likely via its role as cofactor for nitrite reductase.

In the search for new antifungal targets, Barrera et al. (2014) compared fungal and mammalian protein domains as well as protein domain architectures and suggested Met8 as a potential antifungal target due to its presence within the fungal kingdom and absence within mammals. However, our results employing non-neutropenic and neutropenic bronchopulmonary infection models, suggest that siroheme biosynthesis is dispensable for virulence in mice (Figures 5B,C). These data indicate that neither nitrate assimilation nor sulfate assimilation or NO detoxification play a role in pathogenicity of *A. fumigatus* in this host. In agreement, nitrate is not an expected nitrogen source in mammals. Moreover, the wt-like virulence of *A. fumigatus* mutants lacking either the sulfate transporter or sulfite reductase, previously suggested that sulfate assimilation does not play a role in a murine virulence model of *A. fumigatus* (Amich et al., 2016). Furthermore, a mutant, which showed low NO resistance due to lack of cytosolic flavohemoglobin (FhpA), mitochondrial flavohemoglobin (FhpB), *S*-nitrosoglutathione reductase (GnoA), or combinations thereof displayed wt-like pathogenicity in a murine model for invasive pulmonary aspergillosis (Lapp et al., 2014). Consequently, our study supports previous studies by showing that even the combination of the lack of sulfate assimilation, nitrate assimilation, and low NO resistance does not impair virulence in this mammalian host. Remarkably, however, lack of Met8 resulted in mild virulence attenuation in the *G. mellonella* infection model revealing differences in interaction of *A. fumigatus* with *G. mellonella* and mouse. Possible reasons for the different impact of Met8 on virulence in the insect vs. the murine models could be: (i) availability of sulfur sources in the two host niches, (ii) the different temperature of the insect (30°C) compared to the mouse (37°C) models, or (iii) a difference in the role of NO detoxification in the different hosts. However, growth assays on *G. mellonella* extracts and hemolymph did not reveal a significant impact of Met8 (Figure 6A). Moreover, growth assays at different temperatures (Figures 6A–C) did not reveal a particular influence of Met8 in adaptation to temperature. Therefore, it is most likely that NO detoxification is of more importance in the insect compared to the murine models. Notably, an *A. fumigatus* mutant lacking flavohemoglobin-mediated NO detoxification was previously shown to display wt-like virulence in *G. mellonella* (Lapp et al., 2014). However, it might be possible that a defect in nitrite reductase has different consequences with respect to NO detoxification compared to a lack of flavohemoglobins.

## Author Contributions

HH and A-MD conceived and designed the study. A-MD carried out all *in vitro* experiments and wrote the manuscript in consultation with HH, UB, and NO. UB performed the virulence analysis in *Galleria mellonella*. NO and YS performed the virulence analysis in mouse. HH supervised the project.

## Conflict of Interest Statement

The authors declare that the research was conducted in the absence of any commercial or financial relationships that could be construed as a potential conflict of interest.

## Footnotes

1 https://prosite.expasy.org/PDOC00314

2 http://meme-suite.org/doc/fimo.html
